# Reinforcing personalized persuasion in task-oriented virtual sales assistant

**DOI:** 10.1371/journal.pone.0275750

**Published:** 2023-01-05

**Authors:** Aritra Raut, Abhisek Tiwari, Subrata Das, Sriparna Saha, Anutosh Maitra, Roshni Ramnani, Shubhashis Sengupta

**Affiliations:** 1 Dept. of Computer Science, Ramakrishna Mission Vivekananda Educational and Research Institute, Belur, Howrah, India; 2 Dept. of Computer Science and Engineering, Indian Institute of Technology Patna, Patna, Bihar, India; 3 Accenture Labs, Bangalore, Karnataka, India; National Institute of Technology Silchar, India, INDIA

## Abstract

**Purpose:**

Existing task-oriented virtual agents can assist users with simple tasks like ticket booking, hotel reservations, etc. effectively and with high confidence. These virtual assistants, however, assume specific, predictable end-user behavior, such as predefined/servable objectives, which results in conversation failures in challenging situations, such as when goals are unavailable.

**Methodology:**

Inspired by the practice and its efficacy, we propose an end-to-end framework for task-oriented persuasive dialogue generation that combines pre-training and reinforcement learning for generating context-aware persuasive responses. We utilize four novel rewards to improve consistency and repetitiveness in generated responses. Additionally, a meta-learning strategy has also been utilized to make the model parameters better for domain adaptation. Furthermore, we also curate a personalized persuasive dialogue (PPD) corpus, which contains utterance-level intent, slot, sentiment, and persuasion strategy annotation.

**Findings:**

The obtained results and detailed analysis firmly establish the effectiveness of the proposed persuasive virtual assistant over traditional task-oriented virtual assistants. The proposed framework considerably increases the quality of dialogue generation in terms of consistency and repetitiveness. Additionally, our experiment with a few shot and zero-shot settings proves that our meta-learned model learns to quickly adopt new domains with a few or even zero no. of training epochs. It outperforms the non-meta-learning-based approaches keeping the base model constant.

**Originality:**

To the best of our knowledge, this is the first effort to improve a task-oriented virtual agent’s persuasiveness and domain adaptation.

## 1 Introduction

Recent research in natural language processing has focused on developing models for conversational agents, which have many applications ranging from healthcare, business, sales domain, etc. Conversational agents can be of two types based on the nature of the goal: one is a task / goal-oriented virtual agent (virtual agent) [1], and the other is chit chat agent [2]. Chit-chat agents interact with users as companions to satisfy communication needs and create long-term relationships, whereas the former strives to assist users in achieving tasks.

In recent few years, task-oriented conversational agents have been grabbing interest in the domain of the Natural Language Generation. As mentioned earlier, these agents help users solve several tasks like hotel booking, ticket reservation, product purchasing, etc. In a simple task-oriented dialogue generation setting, there are several modules, one DST (dialogue state tracker) for extracting the belief states, one module for searching a query in the database based on the belief states, a policy learning module to determine the suitable action against the context and finally one NLG (Natural Language Generation) module to generate the response. These modules are frequently modeled and assessed independently. The pipeline approach has the obvious disadvantage that error propagation from cascaded components might harm succeeding sub-tasks [3]. So, the appeal for developing some end-to-end systems has increased, and there are few attempts. Some research has generated the system act and response jointly while maintaining ground truth belief states (Chen et al. 2019 [4], Wang et al. 2020 [5]). Some approaches have come close to fully modeling TOD (Task-Oriented Dialogue) agent, but they use different decoders for each component. For example, Lei et al. [6] and Liang et al. [7] generated belief spans and reactions using a seq2seq model. On the other hand, multiple decoders are proposed by Zhang, Ou, and Yu [8] to generate belief spans, act spans, and reactions. This concept is further generalised by SimpleTOD [9] to an end-to-end environment in which belief states are generated in addition to ground truth values. Additionally, they include database results in the training procedure. Then finally, Yang et al. [10] proposed a fully end-to-end dialogue system, UBAR. Based on the context, this model not only extracts belief states but also generates actions and responses on its own. The training objective of UBAR was to maximize the probability of the next word prediction based on the current word.

**Fig 1:**
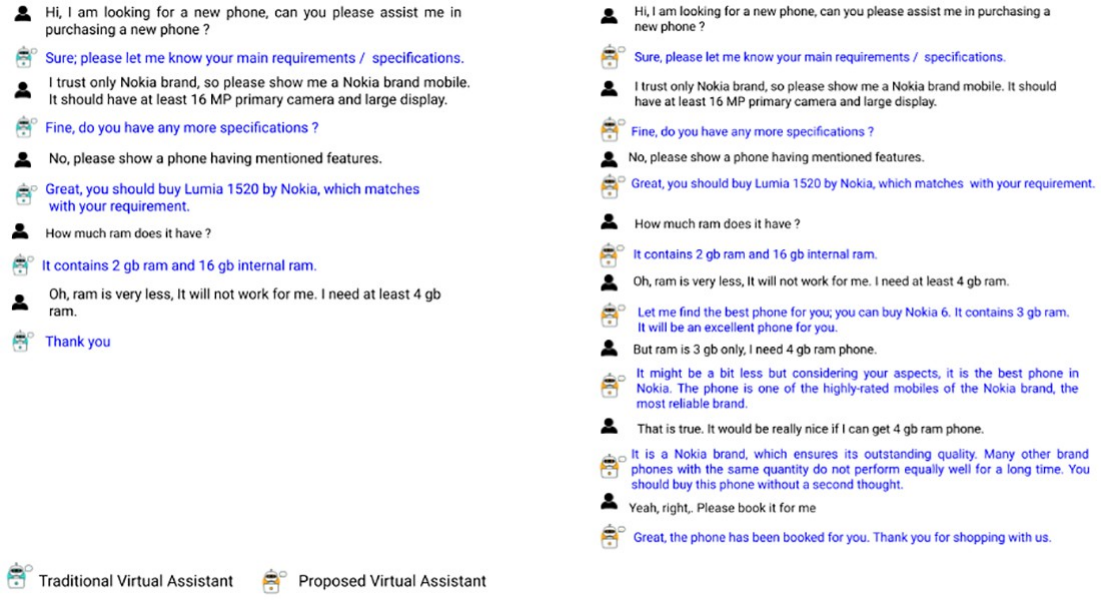
Performance of a traditional virtual agent and proposed agent on a goal unavailability scenario.

In the case of a few specific task domains like sales, it needs to generate some persuasive responses according to the context. In case of goal unavailability or user dissatisfaction, the previous models will fail to fulfill the task in most cases. In this scenario, if we can somehow perform persuasion while keeping the user’s needs in mind, the chance of task completion increases. One example is shown in Fig 1. Even this can be helpful to the user. There might be a different set of products that will fulfill most of the user’s needs, but the user has no information about it. During persuasion, the model may suggest something from those models which can make the user satisfied. According to current research on tailored conversational agents [11, 12], adopting distinct human-oriented chatbot identities or conversational methods can substantially impact user reactions and make the interaction more engaging. These conversational agents considerably improved the user-targeted personalization.

While performing persuasion, there can be a few complex situations, like when the agent gets consecutive negative sentiments from the user. Then it is evident that the kind of response the agent generates does not satisfy the user. Losing consistency with the context or generating the same information again and again while persuading may be the two significant issues in this scenario. To tackle this, we have used an RL(Reinforcement Learning) based reward and penalized the loss. Our reward is a collection of four sub-rewards. Among them, two rewards, context consistency and repetitiveness reward are introduced to address the above mentioned problems. We also have introduced action consistency reward and sentiment-based reward to ensure that the agent generates responses consistent with the chosen action and that the agent satisfies the user with its responses, respectively.

A task-oriented virtual assistant with such a powerful, persuasive skill may benefit various sales environments. For this reason, we must constantly consider an agent’s domain adaptability while developing one. In our case, we have five subdomains: phone, camera, tablet, laptop, and Computer. This can be extended into a few other domains like refrigerator, microwave oven, etc. To make that, we need sufficient data, but we may not collect that, and the high annotation cost inhibits developers from creating their own NLG component from the ground up. Thus, using a fair amount of annotated data to train a language generator module that can be adapted to various other domains or tasks (Which doesn’t have a fair amount of annotated data) is tremendously beneficial. We used a generalized optimization-based meta-learning approach to directly increase the optimization procedure for the low-resource NLG challenge rather than framing the problem as a model-based approach. We found that a recently developed model-agnostic meta-learning algorithm (MAML) [13] is a good match for the low-resource NLG challenge. This MAML aims to learn a better initialization of model parameters that facilitates fast adaptation to new low-resource NLG scenarios.

We have followed the works of Yang et al. [10] and tried to improve its performance in this persuasive dialogue generation setting. In the case of persuasive dialogues, sentiment plays an essential role while generating responses. For instance, Wang et al. [14] included user sentiment to make an effective user-adaptive system. So, we have passed sentiment information as an extra token. We have evaluated and trained the model on our PPD dataset, which contains persuasive dialogues, and the performance after introducing the sentiment token increased over UBAR. Our main contributions are fourfold which are as follows:

1. We develop a large-scale personalized persuasive dialogue corpus annotated with semantic information (intent, slot, sentiment, user persona, and dialogue act) for the e-commerce domain. This data set has dialogues utilizing different persuasion strategies depending on the context.
2. To the best of our knowledge, this is the first work towards building an end-to-end dialogue agent capable of persuading the user in a goal unavailability situation. This module can even follow different persuasion strategies, depending on the context. For example, let’s say a user has come to buy a mobile phone for his daughter, and the model faces some goal conflict. In that case, instead of some logical dialogues, if the model generates emotional or personal persuasive dialogues, that would be more effective.
3. We have infused RL-based rewards with task-oriented end-to-end NLG module UBAR, which helps the model generate more soothing, more consistent, and more appealing responses while performing persuasion.
4. We have experimented with a different training setup of optimization-based meta-learning to make the model parameters better for low resource sub-domain adaptation.

For the reader’s convenience, acronyms often used in this paper are listed in Table 1.

**Table 1:** Accronyms used in the paper.

Acronym	Stands for
PPD	Personalized persuasive dialogue
DST	Dialogue state tracker
NLG	Natural language generation
TOD	Task oriented dialogue
MAML	Model agnostic meta learning
PAML	Persona agnostic meta learning
RNN	Recurrent neural network
LSTM	Long short-term memory
BiLSTM	Bi-directional long short-term memory
GRU	Gated recurrent unit
GPT	Generative pre-training
CNN	Convolutional neural network

## 2 Related work

Our proposed work is mainly striking the areas of personalized persuasive dialogue agents, infusion of RL in dialogue agents, and domain adaptability of task-oriented dialogue agents. So, in this section, we have summarized the relevant works in the subsequent sections.

### Task oriented virtual agent

Many sequence-to-sequence based conversation generation approaches have been suggested in recent years [15], which encode dialogue context using RNN units (LSMT/GRU) and create answers utilizing the encoded information. After that, the use of pre-trained models such as GPT became popular. Large pre-trained language models have outperformed small pre-trained language models on a variety of NLP tasks [16–18], with GPT-2 [19] and GPT-2 [19] is especially good at language generation tasks. GPT-2 [19] has been extended to create responses in chit-chat dialogue [20, 21]. Budzianowski and Vulic (2019) [22], in the task-oriented dialogue domain, first pointed out the ability to fine-tune all essential information in plain text on GPT-2, which drives a line of enhanced and simplified task-oriented dialogue system designs. Then Yang et al. [10] have finally developed the end-to-end task-oriented agent, which had performed really well, outperforming all the previous works in this domain. On the other hand, in a few recent approaches [23, 24], researchers have attempted to close the gap between chit-chat and task-oriented dialogue agents in an effort to make task-oriented discussion more interesting and appealing.

### Persuasive virtual agent

On the other hand, attempts to incorporate persuasion in NLG module had also been made. The Elaboration Likelihood Model (ELM) of Petty and Cacioppo [25] claims that a person’s persuasion is based on changing degrees of thoughts of processing information and persuasive context. The Persuasion Knowledge Model (PKM) proposed by Friestad and Wright proposes that scientific and common persuasion knowledge are interconnected [26]. Furthermore, authors of [27] claimed that combining personal traits with persuasive information might increase a person’s drive to respond to persuasive communications. Then recently, the research [28] proposes a personalized end-to-end task-oriented conversation system that uses a memory network to create attractive and persona-consistent replies. In other recent publications [29–32], the researchers emphasized the DST module to carry out persuasion in task-oriented conversation agents to catch and address dynamic user needs effectively.

### Reinforcement learning on NLG module

**Fig 2:**
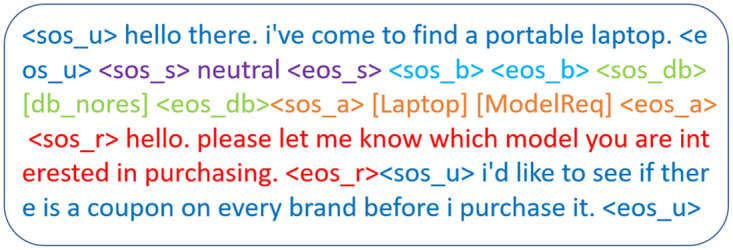
An example of our modified context.

It is difficult to create a personalized conversation agent in supervised learning (SL) framework that can generalize to various users in different settings because of a lack of accessible data and the inherent shifting attitudes and emotions of users in an ongoing dialogue. Because MLE-based models are prone to exposure bias, researchers have recently focused on reinforcement learning (RL) to fine-tune these models because of their capacity to learn from user interactions and improve depending on user input in the form of incentives [33–38]. In a recent work [39], reinforcement learning has also been used to enhance the performance of dialogue generation agent in a different domain, i.e., medical diagnosis. Here in our case, we have taken the idea of following different persuasion strategies depending upon the context and imposed it on the works of Yang et al. [10] to develop an end-to-end persuasive natural dialogue generation module. To enhance the model’s performance, we added a few sub-rewards and changed the context(See Fig 2) a little.

### Meta learning

Meta-learning, also known as learning-to-learn, has recently received a lot of attention. It may be traced back to some early publications [40].“Quick adaptation to fresh and restricted observation data” is a major issue. There are three types of meta-learning that can be used to solve this problem:

1. In **metric-based** meta-learning, the objective is to learn a metric space and then compare low-resource testing samples to high-resource training samples using it. Siamese Network [41], Matching Network [42], Memory-augmented Neural Network (MANN) [43], Prototype Net [44], and Relation Network [45] are some examples of representative works in this domain.
2. In **model based** approach, the concept is to employ a second meta-learner to update the primary learner with a few training instances. Andrychowicz et al., 2016 [46] created an LSTM-based meta learner. For quick model adaptation, Hypernetwork [47], MetaNet [48], and TCML [49] all learn a different set of representations. Ravi and Larochelle [50] suggested an LSTM-based meta-learner to learn the original network’s optimization technique (gradients).
3. The **optimization based** method can be built in such a way that it supports rapid adaptation. By optimizing the gradient towards a good parameter initialization for easy fine-tuning in low-resource scenarios, model agnostic meta-learning [13, 51, 52] achieved state-of-the-art performance. In 2019, Lin et al. [53] used this optimization-based meta-learning to make the model adaptive to new personalities to generate personalized responses in a task-oriented setting. They used meta-learning algorithms to learn multiple personas as separate tasks, which is fundamentally different from optimizing the model to represent all of the personas.

## 3 Problem formulation

We aim to build neural-based goal unavailability adapted virtual assistant that can serve end-users, even in goal unavailability scenarios, and alleviates task failures due to goal conflicts. The agent is also capable of using different persuasion strategies for convincing the user to buy an alternative product. The agent’s response (*R*_*t*_) at time *t*, is being conditioned on user sentiment, belief states, chosen action and is generated as follows:

1. We define context *C*_*t*_ at time step *t* as:
  $$\begin{array}{c} C_{t}=\left\{U_{1},S_{1},B_{1},D_{1},A_{1},R_{1},\ldots ,R_{t-1},U_{t-1},S_{t-1},B_{t-1},D_{t-1},A_{t-1},R_{t-1},U_{t}\right\} \end{array}$$ (1)
  Where *U*_*i*_, *S*_*i*_, *B*_*i*_, *D*_*i*_, *A*_*i*_, *R*_*i*_ stand for user utterance, sentiment, belief states, database query, agent action, and agent response at *i*^*th*^ turn, respectively.
2. The proposed model first encodes(e) the information and generates one token (*R*_*t*_[j]) at each time step depending upon encoded information and previously generated tokens. It can be expressed as follows:
  $$\begin{array}{c} R_{t}=\Pi_{j=1}^{j=n}d\left(e\left(C_{t}\right),R_{t}\left[1:j-1\right]\right) \end{array}$$ (2)
  where, *n* is the number of words in the generated sequence (*R*_*t*_) and *R*_*t*_[*j*] is *j*^*th*^ word of the generated sequence.

## 4 Dataset

We looked at a number of benchmark task-oriented corpora, but we were unable to locate a single dataset that was suitable for the purpose. The properties of several existing conversation datasets are presented in Table 2. In the current work, we have first created a sizable personalised persuasive dialogue corpus called the PPD (personalised persuasive dialogue) corpus. As persuasion is an essential quality of any sales agent, and to encourage researchers to work in the direction of developing some intelligent persuasive conversational agents. The creation of this data collection is intended to hasten the study into creating conversational bots that can persuade users to purchase things when a goal is unavailable. The dataset includes many conversations in which a salesperson tries to persuade a client to buy something, using a variety of persuasive techniques depending on the consumer’s traits and personalities.

**Table 2:** Statistics of the existing datasets and our developed corpus (PPD).

Dataset	Nature	Task	Multi-intent	Dynamic Goal	Task Unavalability	Persuasion	Personalization
ATIS [54]	Task-oriented	Flight booking	✓	×	×	×	×
MultiWoz [55]	Task-oriented	Service booking	✓	×	×	×	×
Persona-Chat [56]	Chit-Chat		×	×	×	×	✓
bAbi [57]	Task-oriented	restaurant reservation	✓	✓	×	×	×
Dear or not [58]	Task-oriented	Negotiation	×	✓	×	✓	×
MMD [59]	Chit-Chat	Fashion assistant	✓	✓	×	×	×
PFG [14]	Task-oriented + Chit-Chat	Donation appeal	×	✓	×	✓	✓
PPD (our dataset)	Task-oriented + Chit-Chat	Electronics assistant (Phone, laptop, and camera booking)	✓	✓	✓	✓	✓

### PPD: Data creation and annotation

Virtual assistants are widely used in commercial applications like online shopping. Thus, for our internal data production, we chose the duty of selling various technological devices. With the help of five mobile retailers, we extensively reviewed the assignment and produced 100 instances of dialogue conversations between sellers and buyers around the work of acquiring electronic items (Mobile, Tab, Camera, Computer, and Laptop). The dialogues that were generated had the following three crucial elements i. Dynamic goal ii. Goal unavailability and iii. Personalized persuasion. The user intent, slot (BIO tag), user sentiment, user personality, persuasive strategy, and dialogue act of each speech in the interaction were also annotated.

### Role of sentiment

Speakers’ responses in conversations are influenced by other speakers’ utterances’ semantic aspects as well as the substance of their own utterances. Sentiment is an example of a feature that subtly conveys feedback and details about the type of action the user wanted to communicate through the message. Sentiment may be efficiently used to track goal conflicts and the results of agents’ persuasion efforts in goal-shifting situations. A consumer may comment, ‘Oh, the colour of the phone is rather drab,’ for example. Here, sentiment (negative) connected to the colour component is the key characteristic that may be used to spot these aim conflicts.

### Role of personalized persuasive strategy

**Fig 3:**
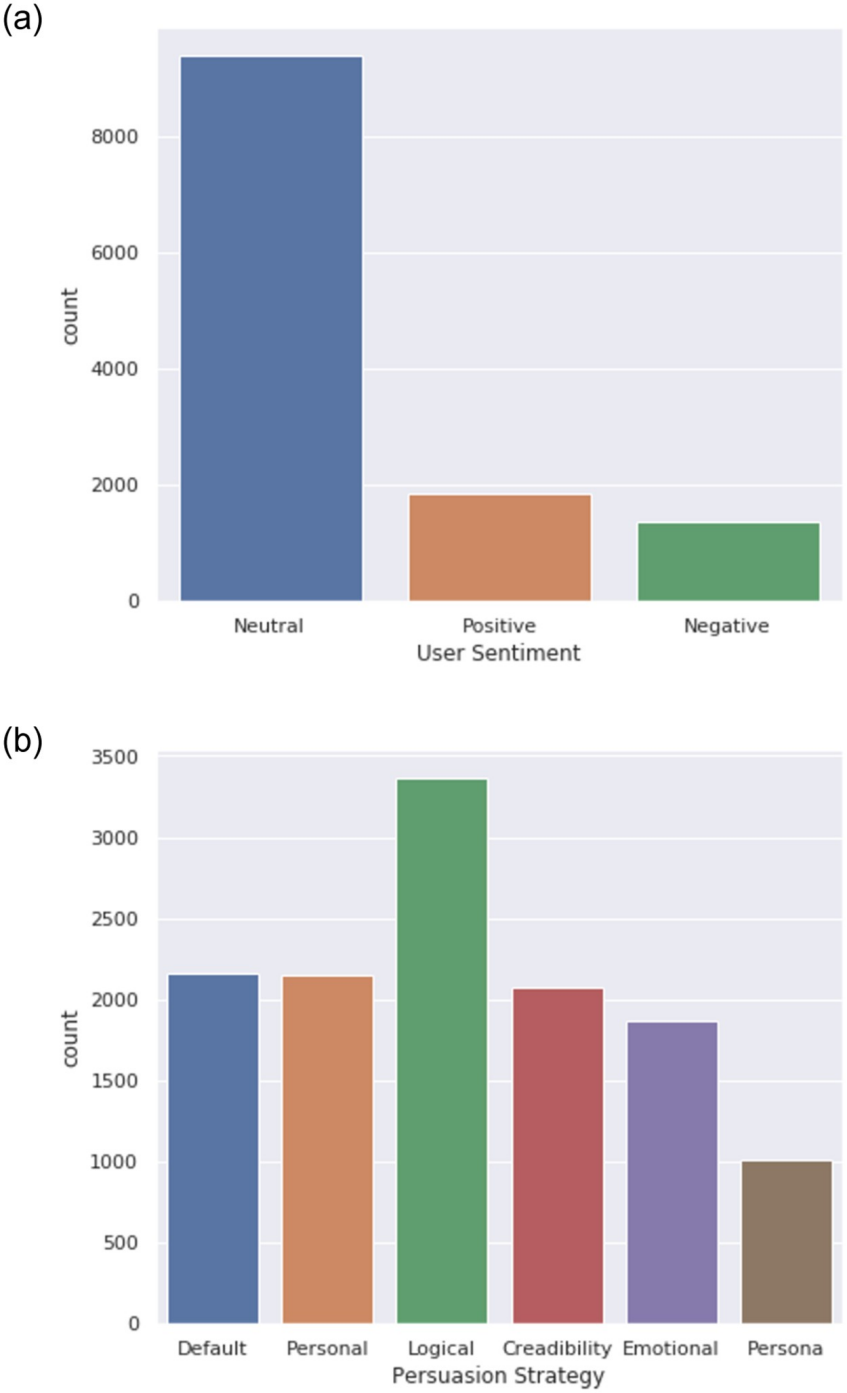
(a) Sentiment and (b) persuasion strategy distribution across PPD corpus.

The effectiveness of persuasion is a very subjective and dynamic issue that much depends on the persuasion target’s relevance and the persuadee’s personality. Even the same persuasion aim and method might not be able to convince the same person in two distinct situations. The suggested approach intends to harness both user personality and dialogue environment for convincing users in goal unavailability scenarios. It is motivated by the importance of customised and dynamic nature of persuasion task. We offer examples of several such ways in Table 3. Distribution of emotion and persuasive tactics within the corpus are shown in Fig 3.

**Table 3:** Examples of different persuasion strategies.

Persuasion strategy	Context (User)	Example
Credibility appeal	I trust only Nokia Brand. So please see something in it.	It is a Nokia brand, which ensures its outstanding quality. Many other brand phones with the same quantity do not perform equally well for a long time. You should buy this phone without a second thought.
Logical appeal	I do not like black color, please find a phone in silver color	You should buy this phone, it has lot of features such as a Radeon Pro 555X G2DDR5 (4 GB) graphic design with Intel Core i7 6 Core processor, 15.4 display size. Its rating is 4.1
Persona-based appeal	It is very costly, see something other.	Sure, but i still highly recommend this phone to you because of its special features particularly gorgeous titan black color.
Personal appeal	No, I do not like this phone. Its storage is less.	This is a great phone, it has received huge number of positive reviews across all countries.
Emotional appeal	Hi, I want to gift a phone to my girl friend on her birthday. She loves photography.	This phone will be perfect gift for a photographer, it has all the features and specifications which are necessary for a photographer. Your girlfriend will love this for sure.

For scaling up the conversational dataset in accordance with example conversations and a full guideline report, we hired five English linguists. For knowledge-based dialogue development, we used GSMArean’s mobile database [60]. A corpus of 1031 conversations and 11602 utterances was produced after they constructed and analysed 931 dialogues. Each speech has been labelled with the appropriate persuasion approach, conversation act, user sentiment, slot, and intent. The kappa coefficient (k), which measures the degree of agreement among annotators on their annotations, was calculated and found to be 0.77, showing a considerable degree of uniform annotation. Table 4 contains statistics from the PPD dataset. In Table 5, we have additionally reported metadata data such as intent and slot lists.

**Table 4:** PPD dataset statistics.

Attribute	Value
Total no. of dialogues	1031
Total no. of utterances	11602
No. of unique words	5937
Avg. dialogue length	11.25
No. of persuasion strategies	6
No. of samples in knowledge base	2697
No. of attributes	18

**Table 5:** Intent, slot, dialogue act, sentiment, and persuasion strategy list of the PPD dataset.

**Intent**	greet, specification, inform, request, persuasion, thanks, preq, done
**Slot**	model, brand, battery, ram, p_camera, s_camera, radio, display_size, status, sim, gps, os, color, internal_ram, weight, released_year, released_month, price, phn_key, specifications, sp_done, features
**Dialogue Act**	greet, specification_request, specification_done, inform, request, result, recommend, persuasion, booking_request, close
**Sentiment**	positive, negative, neutral
**Persuasion Strategy**	Default, Credibility appeal, Logical appeal, Personal appeal, Emotional appeal, Persona based appeal

## 5 Methodology

The work aims to develop a neural-based persuasive dialogue generation framework to deal with goal unavailability scenarios effectively. In the first part of this section we have elaborated the pipeline of UBAR and in the later parts we have discussed how our USBAR model works and what rewards we have introduced to improve the performance.

### 5.1 UBAR pipeline

The pipeline of the UBAR module is very simple. Let’s say, we have our very first user utterance, *U*_0_, at turn *t* = 0. After receiving the user utterance, UBAR generates the components as described below.

**Fig 4:**
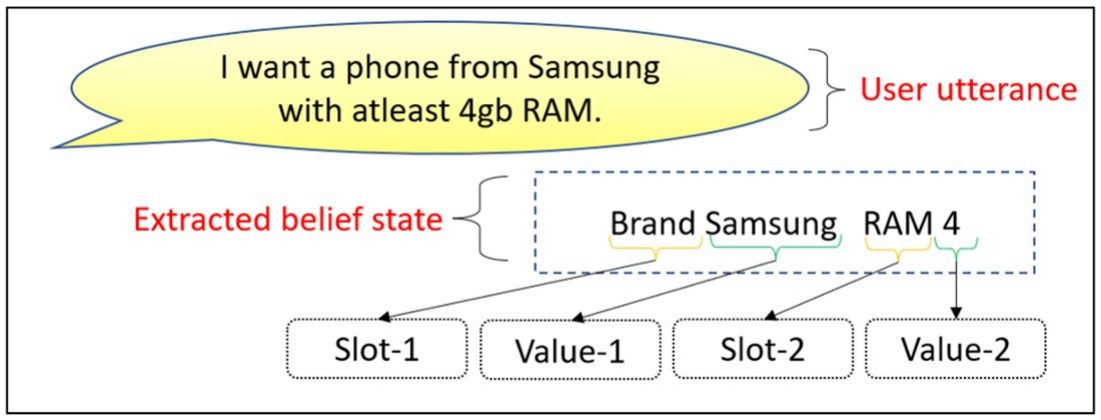
An example of our belief state.

1. The model extracts the belief states *B*_0_, based on *U*_0_. Belief state at each turn is basically a set of decoupled slot-value pairs {*slt*_0_, *v*_0_, *slt*_1_, *v*_1_, ….*slt*_*n*_, *v*_*n*_}, where each pair (*slt*_*i*_, *v*_*i*_) consists of slot and value information extracted from the current utterance. One example of this belief state has been shown in Fig 4.
2. After extracting the belief states *B*_0_, it performs the database query. This provides the number of database instances *D*_0_ matching with the belief states, *B*_0_.
3. Finally based on [*U*_0_, *B*_0_, *D*_0_], it generates agent action *A*_0_, and the delexicalized response *R*_0_. Delexicalized response means, the model is generating special placeholders in the responses for specific slots. For example a brand name in the generated response is <*value*_*brand*>. Later these placeholders should be replaced by the respective values from the database query result. This completes the very first turn.

**Fig 5:**
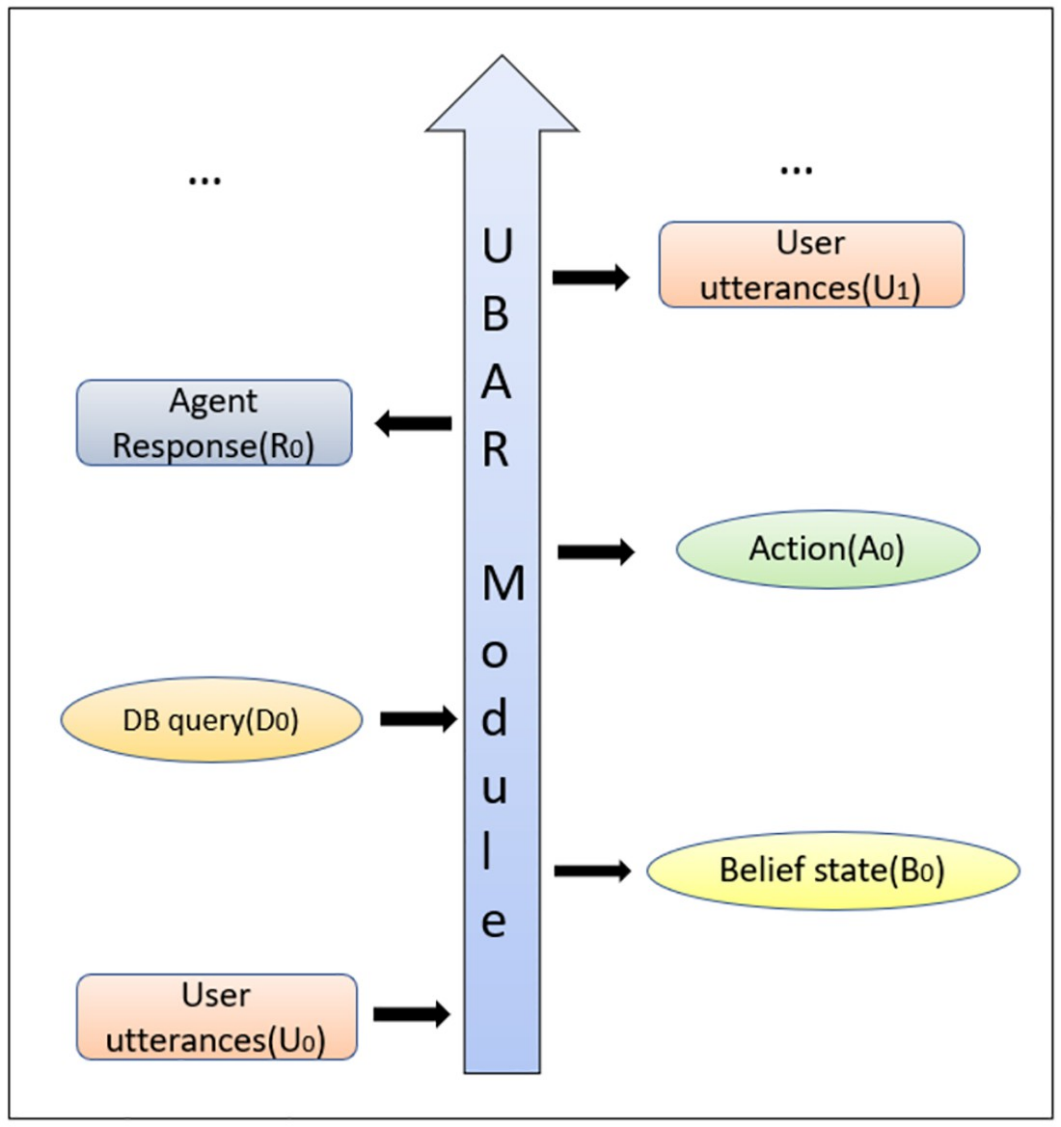
UBAR workflow.

At the next turn (t = 1), again the user will say something (*U*_1_). Now for belief state (*B*_1_) extraction, this *U*_1_ will be concatenated with all the previous contents in order to form the context. The final context for extracting the belief states for this turn will be [*U*_0_, *B*_0_, *D*_0_, *A*_0_, *R*_0_, *U*_1_]. Flow from this point will be exactly as same as the mentioned steps. Similarly at turn *t*, UBAR takes [*U*_0_, *B*_0_, *D*_0_, *A*_0_, *R*_0_, …, *U*_*t*−1_, *B*_*t*−1_, *D*_*t*−1_, *A*_*t*−1_, *R*_*t*−1_, *U*_*t*_] as context and generates *B*_*t*_, *D*_*t*_, *A*_*t*_ & *R*_*t*_, respectively. The overall pipeline of UBAR has been shown in Fig 5.

The previous models used only dialogue history ([*U*_0_, *R*_0_, *U*_1_, *R*_1_, …, *U*_*t*_]) in the context to generate response at turn *t*, while UBAR uses all the previous components (user utterance, belief states, database query instances, actions & agent responses) in the context.

### 5.2 USBAR workflow

**Fig 6:**
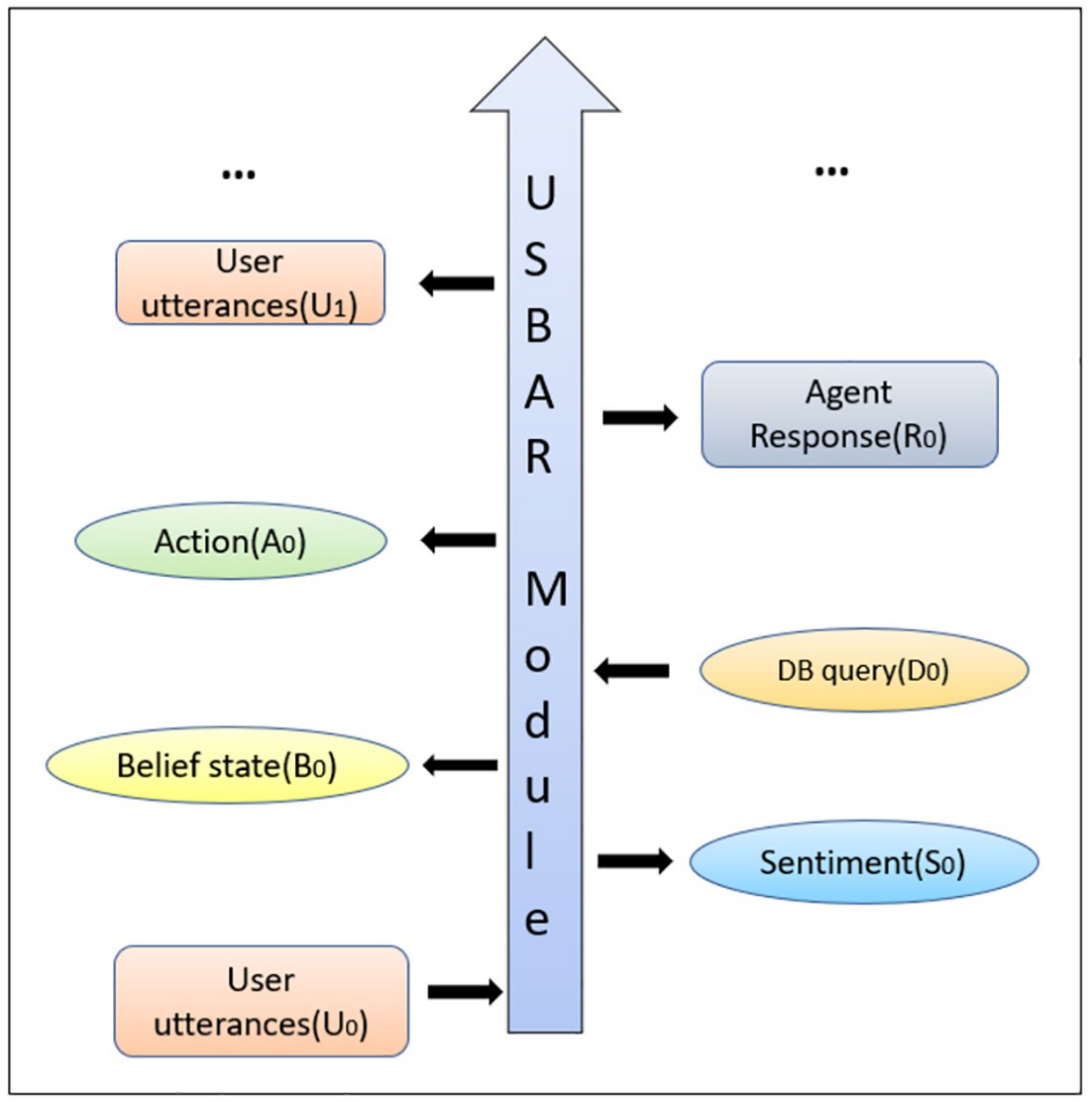
USBAR (our proposed model) workflow.

We have almost maintained the workflow of UBAR (see section 5.1) and introduced the sentiment token as an extra information in the context. At turn *t* = 0, after receiving the user’s turn *U*_0_, flow of USBAR is as follows:

1. The model performs sentiment classification of the user utterance (*U*_0_). Basically it generates a word (positive/negative/neutral) representing the sentiment (*S*_0_) of the user utterance.
2. At this point, our model uses [*U*_0_, *S*_0_] as the context and performs belief state extraction. Belief state *B*_0_ for our model is as same as mentioned in section 5.1.
3. Following UBAR, our model also performs database query based on the belief state *B*_0_, and finds the number of database instances (*D*_0_) matching with *B*_0_.
4. Finally based on [*U*_0_, *S*_0_, *B*_0_, *D*_0_], the model generates required agent action *A*_0_ and delexicalized response *R*_0_. This overall flow is shown in Fig 6.

This flow continues till the end of the conversation.

At the training time, for UBAR we just calculate the cross-entropy loss and update the model parameters accordingly. Following this procedure we have faced a few issues like repetitiveness, lack of consistency with the context and the chosen agent action etc. These problems occur especially when the user expresses negative sentiment at the consecutive turns. This may not lead the conversation the way we want. To tackle these problems, in USBAR, we calculate a reward *r*^*t* = 0^ for the generated response. This reward will make the model aware of the quality of this response. For the next turns, we again follow the previous steps and we keep on calculating rewards for every turn. At the very end of the whole conversation, following UBARs procedure, we calculate a cross-entropy loss, *l*. In addition, we consider the expectation of the rewards (calculated at each turn), in order to have a single reward for the whole conversation, as -

$$\begin{array}{c} r=\frac{\sum_{i=0}^{n}r^{t=i}}{n} \end{array}$$ (3)

Where we assume there are *n* turns in that conversation. We use this reward *R* to calculate our final loss as-

$$\begin{array}{c} L=l+r \end{array}$$ (4)

**Fig 7:**
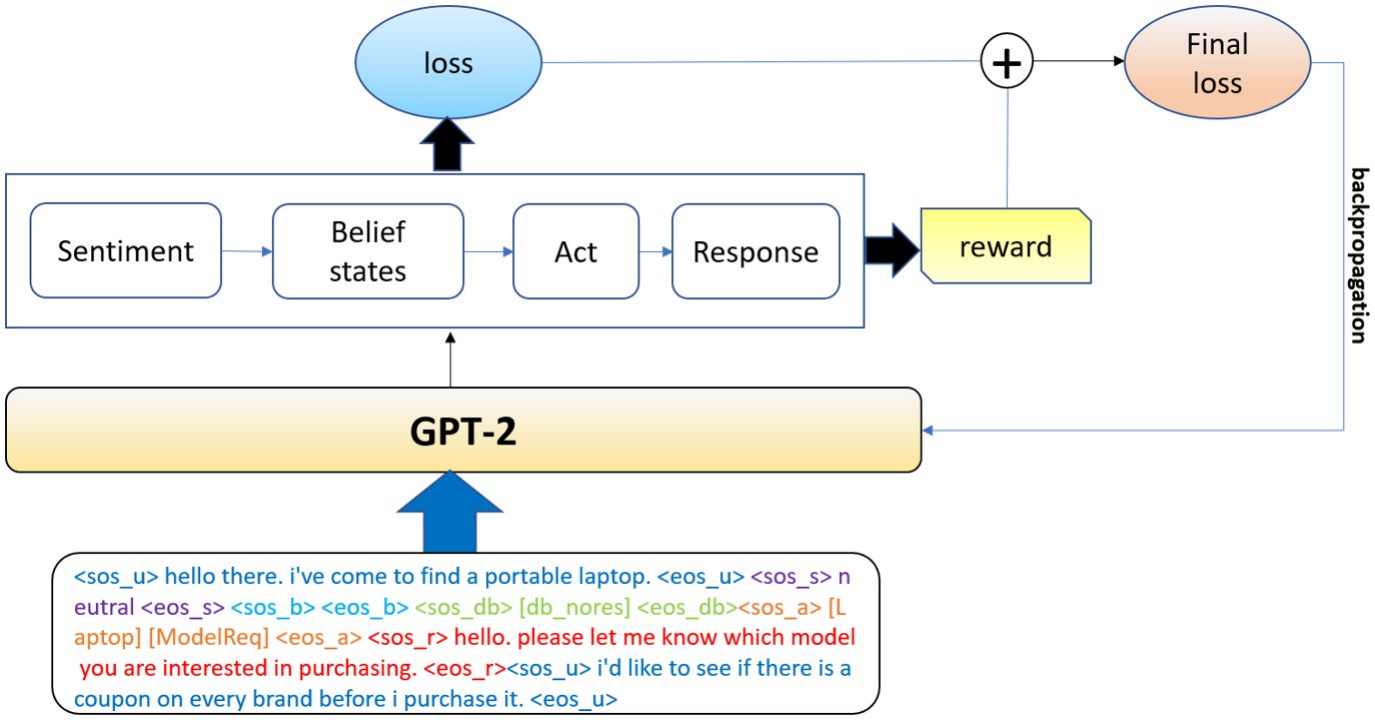
The overall training pipeline.

This penalized loss is used to perform the back propagation in order to update the model parameters. The whole training pipeline has been shown in Fig 7. The details of the rewards are provided in section 5.3.

Other than this, we also have used gradient based meta learning technique to train our model which is discussed in a detailed manner in section 5.5.

### 5.3 Rewards

We have introduced 4 sub-rewards in order to penalize the loss on a session level. These reward are: Repetitiveness reward (*r*_1_), Consistency reward (*r*_2_), Action consistency reward (*r*_3_) and Sentiment based reward (*r*_4_). So, the final reward function is-

$$\begin{array}{c} r=\alpha_{1}r_{1}+\alpha_{2}r_{2}+\alpha_{3}r_{3}+\alpha_{4}r_{4} \end{array}$$ (5)

Then we will penalize the batch loss with these rewards just by adding them. This is just to make sure that we get less repetitiveness and more consistency (both with the context and the chosen action) while generating the responses. The details of these sub-rewards are discussed below.

#### 5.3.1 Repetitiveness reward

According to [61], the models tend to generate more often occurring utterances in the dataset, and this repetition usually occurs at the exact lexical level. As a result, the conversation falls flat and eventually it can affect the persuasion. If the model tries to persuade with the same features of some other product again and again, the user will definitely loose interest. So, to avoid this problem, we use Jaccard Score, a unigram-based measure of similarity between earlier utterances and the current generated response. The sentences are normalised first with spaCy1, and the resulting score is then used as a sub-reward.

$$\begin{array}{c} r_{1}^{t}=\frac{R_{0}\cap R_{1}\cap \ldots \cap R_{t}}{R_{0}\cup R_{1}\cup \ldots \cup R_{t}} \end{array}$$ (6)

#### 5.3.2 Consistency reward

We use **Meteor score** [62], a machine translation evaluation metric based on a generalised idea of unigram matching between machine-produced and human-produced reference translations. Here we determine **Meteor score** [62] between the generated responses (hypothesis) and the gold human response in order to generate human-like responses (reference). We chose the golden human response as a benchmark for assessing its resemblance to our generated responses since we believe it is optimally consistent with the dialogue. Meteor calculates a score for this matching using a combination of unigram-precision, unigram-recall, and a measure of fragmentation that is designed to directly capture how well-ordered the matched words in the machine translation are in relation to the reference once all generalized unigrams matching between the two strings have been found. Meteor score was chosen because it employs WordNet to find synonyms when exact matches aren’t found [63], and it has a high connection with human assessment in machine translation jobs.

#### 5.3.3 Action consistency reward

Consistency between the generated response and the chosen action is very much important, especially in case of different persuasion strategies. For example let’s say, for a particular turn in a conversation the chosen action is emotional appeal, but the model is trying to convince the user with some features in a logical manner. That may lead the conversation to an end. It is absolutely necessary to maintain consistency with the chosen action. To make it sure that the generated response is consistent with the chosen agent action, we have introduced this sub-reward.

To calculate this reward we need to have the probability distribution of the generated response over the action classes (different persuasion strategies are included as actions), and then we can calculate this sub-reward as:

$$\begin{array}{c} r_{3}=P_{s_{j}}^{R,t}-\beta \sum_{i\in S\backslash \{s_{j}\}}P_{s_{i}}^{R,t} \end{array}$$ (7)

Where, $P_{s_{i}}^{R,t}$ denotes the probability of the response *R* at turn *t* belonging to the *i*^*th*^ action class. Here in this equation, *j*^*th*^ class is the ground truth action class given in that context. To get the probability distribution over all the action classes, we built an action or strategy classifier (mentioned in section 5.4) by fine tuning RoBERTa, which achieved an overall accuracy of 82% and macro F1 score of 68.56.

#### 5.3.4 Sentiment based reward

The main motive of persuasion is to make the user satisfied. In other words, the model should make sure that the user doesn’t express negative sentiment consecutively. Because if the user is showing negative sentiment again and again, that means, the same kind of response is getting generated which is not working at all. In that scenario, the model must understand that it has to change the previous persuasive strategy or it should stop persuading and recommend some other products to the user. So, to capture this, the idea of this sub-reward is very simple. If the user’s sentiment is negative in consecutive 3 or more turns, a penalty of 1 is added. Let’s say, we get negative sentiment from the user for consecutive 4 times, then the penalty would be 2(= 1+ 1). Then let’s say after that we got some positive or neutral sentiments and then again we get consecutive 3 negative sentiments. Then the penalty would be 3(= 2+ 1).

### 5.4 Action classifier

**Fig 8:**
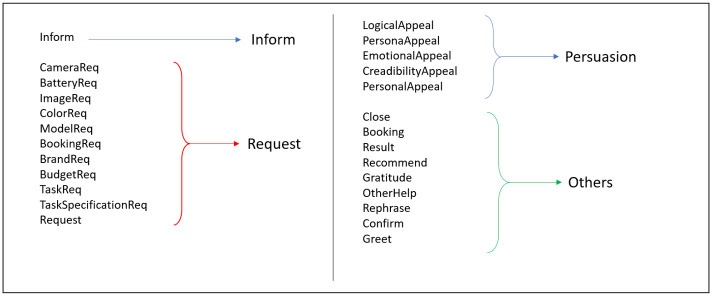
26 action class grouping for the hierarchical action classifier.

Here we have 26 different kinds of actions including 5 main different persuasion strategies (Personal appeal, Persona appeal, Logical appeal, Emotional appeal and Credibility appeal), excluding ‘Default’ persuasion strategy. Our model chooses one from them at every turn, depending upon the context. We have tried a few models like BiLSTM, CNN and RoBERTa [64] to perform this task. We have failed to achieve an accuracy beyond 60 percent, so we have gone for the hierarchical approach. We have divided all the classes into main four classes- **inform, request, persuasion** and **others** and then divided the persuasion class into another 5 sub-classes representing the different persuasion strategies (details provided in Fig 8). We have experimented with different window sizes of context and using RoBERTa, we obtained the best classifier in terms of accuracy and macro F1 score. The metric, accuracy is defined as:

$$\begin{array}{c} Accuracy=\frac{no.\;of\;correct\;prediction}{total\;no.\;of\;instances} \end{array}$$ (8)

On the other hand, using the arithmetic mean (aka the unweighted mean) of all the per-class F1 scores, the macro-averaged F1 score (or macro F1 score) is calculated. Where F1 score for each class is defined as:

$$\begin{array}{c} F1\;score=\frac{True\;positive}{True\;positive+1/2(False\;negative+False\;positive)} \end{array}$$ (9)

The detailed results are presented in Tables 6 and 7.

**Table 6:** Performances of action classifier for main 4 classes.

Models	Window size	Accuracy	Macro F1 score
**CNN**	1	78.30	69.54
	3	80.78	71.32
	6	83.14	73.36
**Bi-LSTM**	1	80.20	74.35
	3	82.39	76.65
	6	85.13	78.34
**RoBERTa**	1	**83.89**	**78.23**
	3	**84.50**	**78.67**
	6	**88.65**	**80.56**

**Table 7:** Performances of action classifier for 5 persuasive classes.

Models	Window size	Accuracy	Macro F1 score
**CNN**	1	49.24	41.83
	3	53.28	42.45
	6	55.14	44.33
**Bi-LSTM**	1	50.21	45.45
	3	53.54	46.62
	6	56.13	45.55
**RoBERTa**	1	**59.67**	**53.22**
	3	**65.78**	**54.37**
	6	**68.56**	**60.54**

### 5.5 Meta learning

**Algorithm of Meta Learning for domain adaptation**

**Require:** *D*_*train*_

**Require:** *α*_*meta*_, *β*_*meta*_: step size hyperparameters

1: Randomly initialize *θ*

2: **while** not done **do**

3:  Sample batch of different sub-domain $D_{d_{i}}\in D_{train}$

4:  **For all** $D_{d_{i}}$ **do**

5:   $\left(D_{d_{i}}^{train},D_{d_{i}}^{valid}\right)\in D_{d_{i}}$

6:   $\theta^{{}^{\prime }}=\theta -\alpha_{meta}\nabla_{\theta }L_{D_{d_{i}}^{train}}\left(f_{\theta }\right)$

7:  **end for**

8:  $\theta =\theta -\beta_{meta}\sum_{D_{d_{i}}\in D_{train}}\nabla_{\theta }L_{D_{d_{i}}^{train}}\left(f_{\theta_{d_{i}}^{{}^{\prime }}}\right)$

9: **end while**

**Fig 9:**
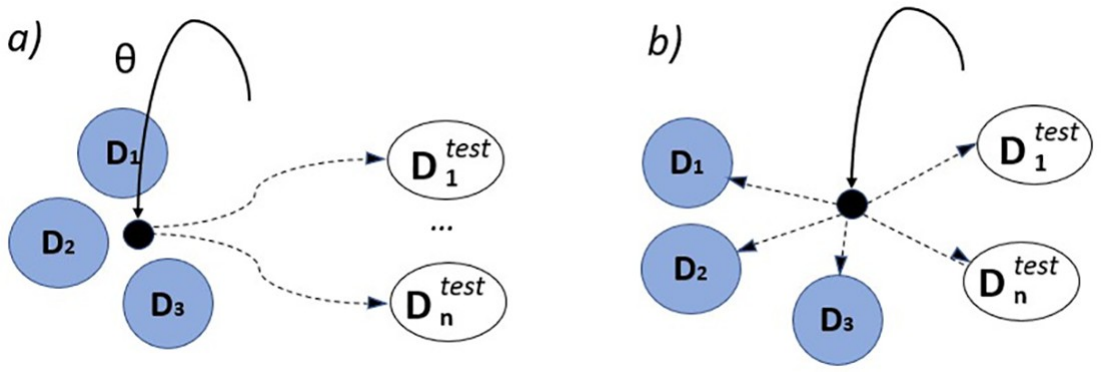
The difference between finetuning from a) joint training on all sub-domains and b) meta-learning sub-domain. The solid line represents the optimization path of the initial parameters and dashed line the fine-tuning path. Meta-learned initial parameters can faster adapt to a new sub-domain.

To extend this work into some other unseen sub-domains like refrigerator, air condition, micro-wave oven etc. we need to make the parameters easily adoptable. Following the PAML(Persona Agnostic Meta Learning) [53], we use meta-learning algorithm to learn different sub-domains as separate tasks, which is fundamentally different from optimising the model to represent all of the sub-domains. A high-level intuition of the difference between these two approaches is shown in Fig 9. Then we define the sub-domain meta-dataset $D=\{D_{d_{1}},D_{d_{2}},\ldots ,D_{d_{m}}\}$, where *m* is the no. of different sub-domains we have (here, we have merged computer & laptop domains to make it a 4 sub-domain meta-data). Before training, we divide the dataset into two parts, *D*_*train*_ & *D*_*test*_. For each training epoch, we uniformly sample a set of conversations from each $D_{d_{i}}\in D_{train}$, as $D_{d_{i}}^{train}$ & $D_{d_{i}}^{valid}$. After *t* iterations on *D*_*train*_, the model *f*_*θ*_ parameterized by *θ* is updated to $\theta^{{}^{\prime }}$ by standard gradient descent.

$$\begin{array}{c} \theta^{{}^{\prime }}=\theta -\alpha_{meta}\nabla_{\theta }L_{D_{d_{i}}^{train}}\left(f_{\theta }\right) \end{array}$$ (10)

Where, *α*_*meta*_ is the learning rate of inner optimisation and $L_{D_{d_{i}}^{train}}$ is the training loss. Then the model is updated such that it maximizes the log-likelihood for the unseen dialogues, i.e., $D_{d_{i}}^{valid}$. We apply again stochastic gradient descent on the meta-model parameters, *θ*, by computing the gradient of $L_{D_{d_{i}}^{valid}}\left(f_{\theta^{{}^{\prime }}}\right)$, that is-

$$\begin{array}{c} \theta =\theta -\beta_{meta}\sum_{D_{d_{i}}\in D_{train}}\nabla_{\theta }L_{D_{d_{i}}^{valid}}\left(f_{\theta_{d_{i}}^{{}^{\prime }}}\right) \end{array}$$ (11)

where *β*_*meta*_ is meta learning rate. Second order optimization partial derivatives are required for this procedure, which can be generated using any automatic differentiation library (e.g., PyTorch, Tensorflow, etc.). The overall algorithm is shown above.

## 6 Training setup & implementation details

We have implemented our model with HuggingFace’s Transformers [65] and DistilGPT2 [66], a distilled version of GPT-2, in a session level; this means the whole conversation has been passed to the model, and it learns to generate or guess the next word based on the current word. We have used cross-entropy function as our loss, AdamW as our optimizer and standard greedy decoding method with temperature of 0.7. We have calculated the respective rewards at every iteration and added them with the loss, and then this penalized loss has been used to update the respective parameters. We have used *α*_1_ = 1, *α*_2_ = −1, *α*_3_ = −1 and *α*_4_ = 1 in Eq 5 and *β* = 1 in Eq 7 and a batch size of 2 at every iteration.

## 7 Results

We have evaluated our model with different setups on our developed PPD dataset. For automatic evaluation, we calculated BLEU (BiLingual Evaluation Understudy) score [67] and Rouge score. We also have performed human evaluation of this model in terms of repetitiveness, consistency, personalized persuasion and grammatical correctness. The metrics are defined as follows:

- **Repetitiveness:** It measures how much similar the generated responses are. We have defined it as the Jaccard Similarity (see Eq 6) between agent responses in a single conversation.
- **Consistency:** This is defined as the number of slots fulfilled by the agent / number of slots asked by the user.
- **Personalized persuasion:** These were marked on the degree of perceived personalization tactic executed by the agent. On a scale of 5, the agent responses which were able to use the contextual information that was provided by the user at the start of the conversation were given relatively higher points. Expectedly, a neutral response was given 2.5 points.
- **Grammatical Correctness Score (G.C Score):** It measures how good the generated sentence is in terms of grammatical correctness. For each conversation, it is calculated as: Grammatical Correctness = number of grammatically correct responses / total number of turns. Then, finally we have taken the mean over all the conversations in order to get the final Grammatical Correctness score (G.C Score).

We evaluated the performance of our model (USBAR) in comparison to two established techniques, such as UBAR [10] and SimpleTOD [9], which is also a GPT-2-based technique trained on turn-level data without generated belief state and system act in dialogue history. Additionally, we have experimented with altering the base model in the USBAR configuration from DistilGPT2 to DialoGPT [20]. We have fine-tuned models for 50 epochs each and experimented with different settings. For each model we have experimented with a combination of true and generated belief states and actions and measured the performances in terms of the automatic evaluation metrics. We also have measured the performances of each model in terms of our aforementioned human evaluation metrics, but only in an end-to-end setting (using generated belief states and actions in the context). Automatic evaluation results are shown in Table 8 and the human evaluation results are shown in Table 9.

**Table 8:** Automatic evaluation results with different setup.

Models	True bs	True act	BLEU-1	BLEU-2	BLEU-3	BLEU-4	Rouge-1	Rouge-L
**SimpleTOD**	✓	✓	0.25	0.12	0.06	0.01	0.30	0.24
	✓	×	0.23	0.10	0.06	0.01	0.28	0.23
	×	✓	0.16	0.06	0.04	0.01	0.25	0.22
	×	×	0.12	0.04	0.02	0.01	0.20	0.18
**UBAR**	✓	✓	0.28	0.15	0.08	0.04	0.32	0.28
	✓	×	0.26	0.16	0.08	0.02	0.32	0.28
	×	✓	0.24	0.12	0.06	0.04	0.30	0.24
	×	×	0.15	0.08	0.04	0.01	0.24	0.22
**UBAR+r_1_ + r_2_**	✓	✓	0.32	0.16	0.14	0.08	0.40	**0.38**
	✓	×	0.28	0.16	0.10	0.04	0.38	**0.36**
	×	✓	0.25	0.14	0.10	0.04	0.35	0.33
	×	×	0.17	0.08	0.04	0.02	0.26	0.24
**UBAR+r_1_ + r_2_ + r_3_ + r_4_**	✓	✓	0.32	0.18	0.12	0.08	0.36	0.34
	✓	×	0.27	0.16	**0.12**	0.06	0.38	**0.36**
	×	✓	0.26	0.15	**0.11**	0.04	**0.36**	**0.34**
	×	×	0.20	0.10	0.06	0.02	0.29	0.25
**USBAR with DialoGPT**	✓	✓	0.27	0.13	0.08	0.06	0.34	0.32
	✓	×	0.24	0.11	0.08	0.05	0.32	0.30
	×	✓	0.19	0.09	0.06	0.02	0.28	0.26
	×	×	0.16	0.06	0.04	0.01	0.23	0.20
**USBAR**	✓	✓	0.28	0.16	0.10	0.06	0.35	0.30
	✓	×	0.28	0.16	0.08	0.06	0.34	0.28
	×	✓	0.26	**0.16**	0.10	**0.06**	0.32	0.28
	×	×	0.18	0.10	0.06	**0.04**	0.26	0.22
**USBAR+r_1_ + r_2_**	✓	✓	**0.34**	**0.20**	**0.16**	**0.12**	**0.42**	**0.38**
	✓	×	**0.32**	0.16	0.10	0.08	0.38	**0.36**
	×	✓	**0.28**	**0.16**	0.10	**0.06**	**0.36**	**0.34**
	×	×	0.22	**0.12**	**0.08**	**0.04**	0.32	0.24
**USBAR+r_1_ + r_2_ + r_3_ + r_4_**	✓	✓	0.33	0.18	0.14	0.08	**0.42**	**0.38**
	✓	×	**0.32**	**0.18**	**0.12**	**0.10**	**0.40**	**0.36**
	×	✓	**0.28**	**0.16**	0.10	0.04	0.34	0.28
	×	×	**0.24**	**0.12**	**0.08**	**0.04**	**0.34**	**0.30**

*r*_*i*_ stands for respective rewards described in section 5.3, bs = belief state, act = action

**Table 9:** Human evaluation results in an end-to-end setting.

Models	Consistency	Repetitiveness	Personalized persuasion	G.C Score
**SimpleTOD**	0.62	0.40	2.08	0.862
**UBAR**	0.67	0.35	2.31	0.873
**UBAR+r_1_ + r_2_**	0.71	0.29	2.66	0.901
**UBAR+r_1_ + r_2_ + r_3_ + r_4_**	0.71	0.29	2.78	0.915
**USBAR with DialoGPT**	0.67	0.36	2.30	0.875
**USBAR**	0.69	0.33	2.33	0.913
**USBAR+r_1_ + r_2_**	**0.74**	0.27	2.87	0.943
**USBAR+r_1_ + r_2_ + r_3_ + r_4_**	**0.74**	**0.26**	**2.93**	**0.956**

All generated components (sentiment+belief state+agent action) were used in the context

### 7.1 Results without rewards

A careful inspection of results attained by SimpleTOD, UBAR and USBAR(our model) as shown in Table 8 reveals a clear performance improvement in each setting. The improvement is not huge but with the inclusion of a very small information like sentiment in the context, an improvement over UBAR is achieved. The improvement is reflected both in terms of automatic and human evaluation (see Table 9) metrics. We also experimented by changing the pre-trained model to DialoGPT from DistilGPT2 and it is not providing us a greater performance in any of the cases.

### 7.2 Results with *r*_1_ & *r*_2_

These rewards were used mainly to improve the performance in terms of repetitiveness and consistency with the context. After the inclusion of these 2 rewards we can notice a significant rise in the performance both in terms of automatic and human evaluation metrics. Specially there were two human evaluation metrics (repetitiveness and consistency) designed to capture the performance after the inclusion of these two rewards. In those columns of Table 9 also, we can see a significant amount of rise in the performance. That signifies, our motive behind introducing these two rewards is successful.

### 7.3 Results using *r*_1_, *r*_2_, *r*_3_ & *r*_4_

After inclusion of all the rewards, we are not getting a significant improvement over the previous model (with 1st and 2nd reward only). We achieved a good amount of improvement in terms of grammatical correctness and personalized persuasion. The action classifier we designed is not perfect, which is restricting us from getting an significant improvement in case of UBAR. On the other hand, if we look at the case of USBAR, we can see somewhere the performance has gone down after introducing 3rd and 4th rewards over the first 2 rewards. The classifier we are using was not trained with a context which contains sentiment. So in case of USBAR the classifier is becoming more confused. As a result whenever we are using ground truth actions, the model is getting confused and generating some responses which are different than the ground truth. On the other hand whenever the concern is action choosing, the USBAR model is choosing the action such a way that it generates responses which are more closer to the gold human responses. As a result, a performance comparison between USBAR+*R*_1_+*R*_2_ and USBAR+*R*_1_+*R*_2_+*R*_3_+*R*_4_ reveals that relying on generated actions leads to an improved result from the later model but addition of ground truth actions in the context reverses the result. Anyway in an end-to-end setting, we are getting the best result in terms of almost all the metrics (including both human and automatic evaluation metrics), from USBAR+*R*_1_+*R*_2_+*R*_3_+*R*_4_ module.

**Fig 10:**
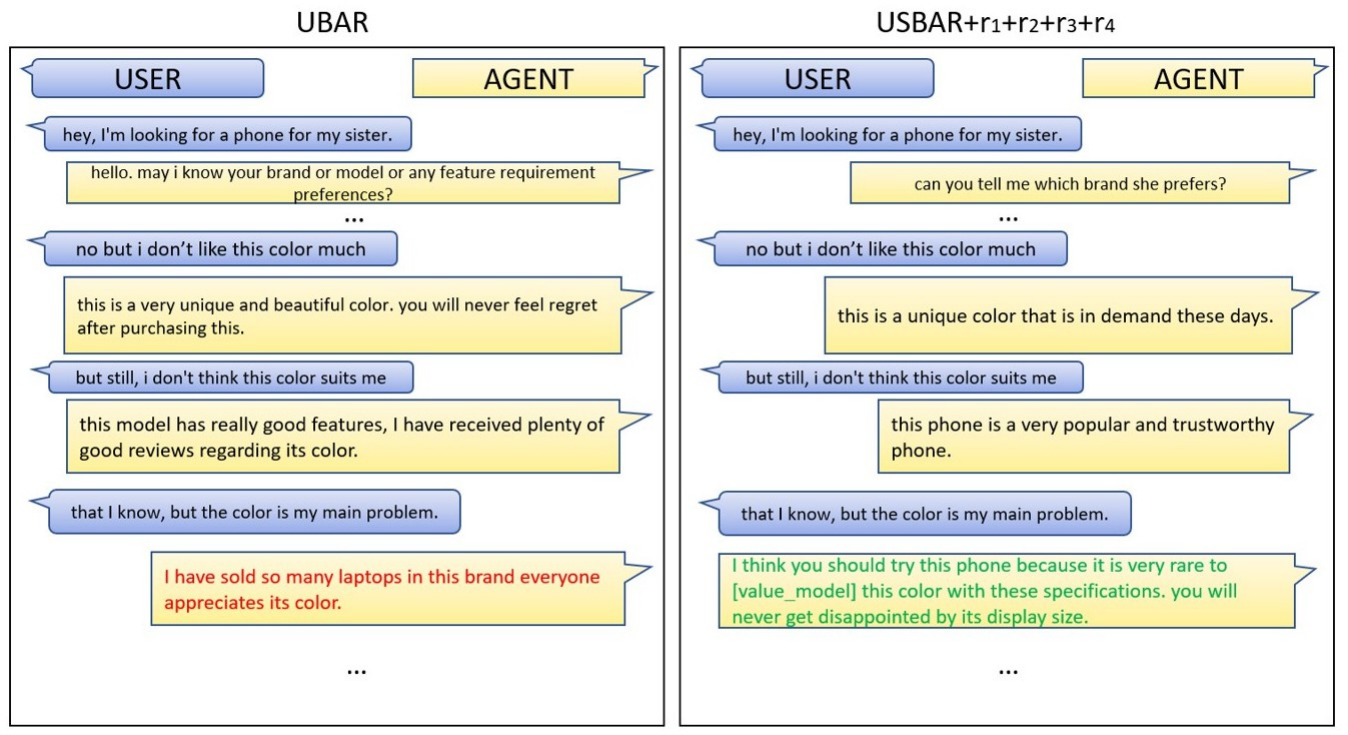
Outputs generated from two different models:(a) output from simple UBAR (b) output from our USBAR+rewards (for simplicity the intermediate components, i.e., sentiment, belief states, db query, agent action, have been omitted).

A comparison between the performances of UBAR and our final USBAR+rewards module is shown in Fig 10. A close look at the left image which is from the UBAR module, reveals that at some point of conversation when the user is showing negative sentiment for consecutive 3rd time the model has clearly lost the context. It is trying to persuade mentioning some different sub-domains. In the same situation, our USBAR module, trained with the reinforcement learning based rewards(right image), is able to catch the context correctly and even after getting three consecutive negative sentiments, it is able to persuade with proper information. One more thing to notice is in the left image, the model is passing the same message in different forms, which can eventually make the user disgusted. In the later case it is changing the strategy. On the second consecutive negative sentiment, it is trying to persuade the user with the brand then again getting back to the uniqueness of the color. This is possibly a better approach. So our final model has clearly achieved an improvement over UBAR. A few more situations are covered in section 10.

#### Meta learning

For the meta learning, we kept each of the domains (except phone, as it has a good amount of data) out at each time, and fine-tuned the DistilGPT-2 for 2000 iterations, following the earlier mentioned algorithm. At each iteration, we picked 4 instances from each of the training domains selected, 2 for training and 2 for validation. Then loaded these parameters in order to fine tune them on the domain on which we are trying to test the domain adaptation. This time we have fine tuned the model for 25 epochs only and measured the performances. We have experimented with few shot (trained for 25 epochs) and zero shot setting. The results for domain adaptation experiments are shown in Table 10

**Table 10:** Domain adaptation results using Meta learning.

Models	Domain	Setting	B-1	B-2	B-3	B-4	R-1	R-L	Con	Rep
**USBAR**	**Tablet**	**Zero shot**	0.10	**0.04**	**0.03**	0.00	0.19	0.16	0.35	0.40
		**few shot**	0.18	0.06	**0.04**	**0.01**	0.26	0.22	0.49	**0.34**
	**Camera**	**Zero shot**	0.16	0.06	0.02	0.00	0.24	**0.20**	0.30	**0.40**
		**few shot**	**0.21**	0.09	0.04	**0.01**	**0.28**	**0.24**	**0.44**	**0.37**
	**Laptop**	**Zero shot**	0.10	0.02	0.00	0.00	0.16	0.13	0.33	**0.39**
		**few shot**	0.18	0.06	0.02	**0.01**	0.24	**0.18**	0.41	**0.36**
**USBAR+MAML**	**Tablet**	**Zero shot**	**0.12**	**0.04**	**0.03**	**0.01**	**0.21**	**0.18**	**0.38**	**0.36**
		**few shot**	**0.20**	**0.10**	**0.04**	**0.01**	**0.28**	**0.23**	**0.51**	**0.34**
	**Camera**	**Zero shot**	**0.18**	**0.08**	**0.04**	**0.01**	**0.26**	**0.20**	**0.32**	0.41
		**few shot**	**0.21**	**0.10**	**0.05**	**0.01**	0.27	0.23	**0.44**	0.38
	**Laptop**	**Zero shot**	**0.13**	**0.04**	**0.02**	0.00	**0.19**	**0.15**	**0.41**	**0.39**
		**few shot**	**0.22**	**0.08**	**0.03**	**0.01**	**0.26**	**0.18**	**0.46**	0.38

Domain stands for the specific sub-domain which was kept out, B-i stands for BLEU-i, R-j stands for Rouge-j, Con stands for consistency, and Rep stands for repetitiveness

## 8 Error analysis

We observed the following two key issues with the proposed model.

- The model is getting confused between different persuasive strategies. Let’s say, the chosen action is emotional appeal but the generated response is not emotional appeal at all. We tried to resolve this problem by our 3rd reward, but the action classifier we made is not enough accurate, especially for different persuasive appeals. A more accurate classifier can help in avoiding this problem totally.
- It is very important for our model to be able to generate correct delexicalized responses. In some scenarios we noticed that our model is unable to generate placeholders for some slots. We have not done slot-value annotations for agent responses in the dataset. The model is fully dependent on the slot-value annotations of user utterances, to learn the placeholders for respective slots, but there are few slot values (like processor, release date, etc.), which rarely appear at the user utterances. As a result, naturally the model fails to learn the proper placeholders for those slots.

## 9 Advantage & limitation

As mentioned earlier, when the end user’s goal is unavailable, the sales field faces its most difficult predicament. Unlike the existing models, the agent we suggest will be effective in trying to convince the user or recommend something else that they might enjoy, rather than failing in such circumstances. In some situations when the model opts for recommendation over persuasion, it makes a suggestion simply by disregarding the most recent specification (belief state) that the user has provided rather than drawing on the strength of a separate recommendation system.

## 10 Case studies

Here we are showing a few more examples of how the two models (UBAR and USBAR+reward) generate responses in different situations. On the left, we are keeping the responses from UBAR and on the right we are keeping the responses from USBAR+rewards.

### 10.1 Generated sample 1

**Fig 11:**
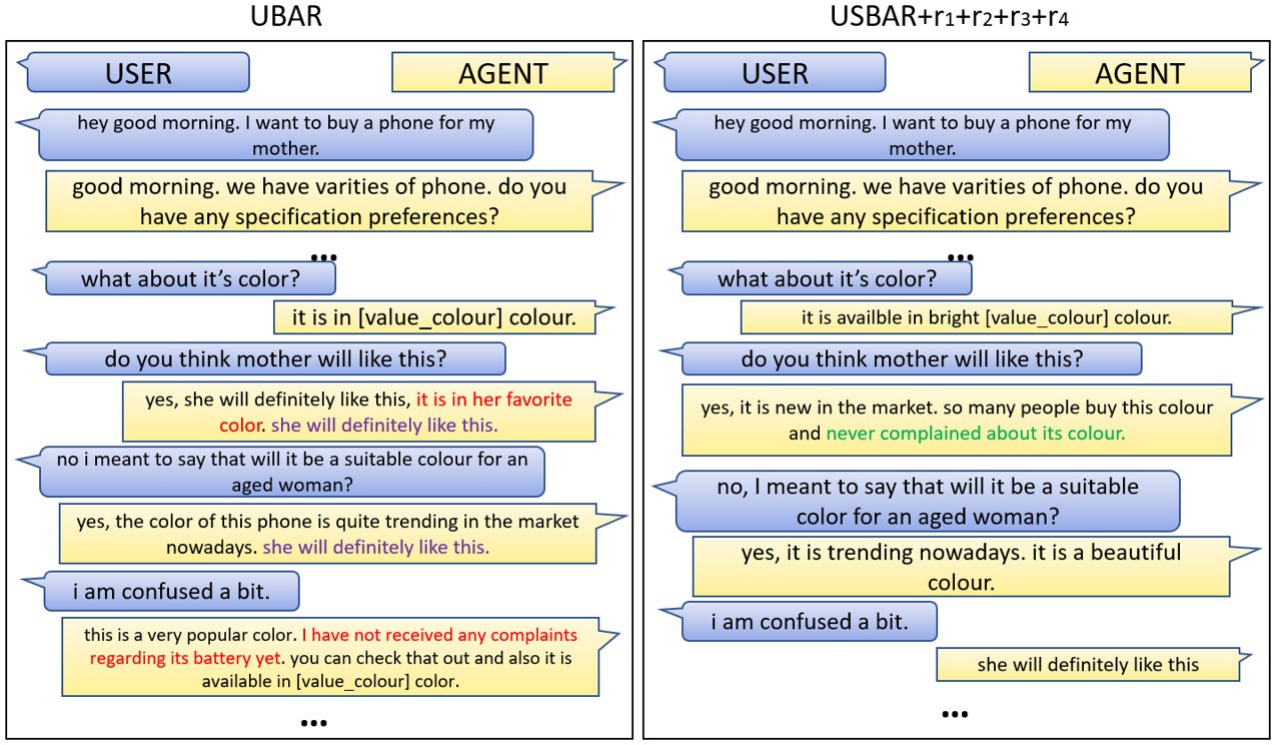
Sample-1.

In this image (Fig 11) we can see that the user is not happy about the colour. He/she is consecutively throwing negative sentiment about it and the model is trying to convince him/her. On the UBAR response (left image), we see at some point the model is saying“… it is in her favorite color…”. The model doesn’t have any previous knowledge about the person for whom the product is getting purchased, or even the user has never gave him that information. So this sentence may somehow mislead the conversation. In addition to that, surprisingly in the last reply, the model is trying to convince the user by saying that it has never received any complaint about its battery. So it is clearly loosing context and as a result the response is not expected at all. Whereas the USBAR+Rewards module is not loosing context and continues persuasion till the end, keeping consistency with the context.

### 10.2 Generated sample 2

**Fig 12:**
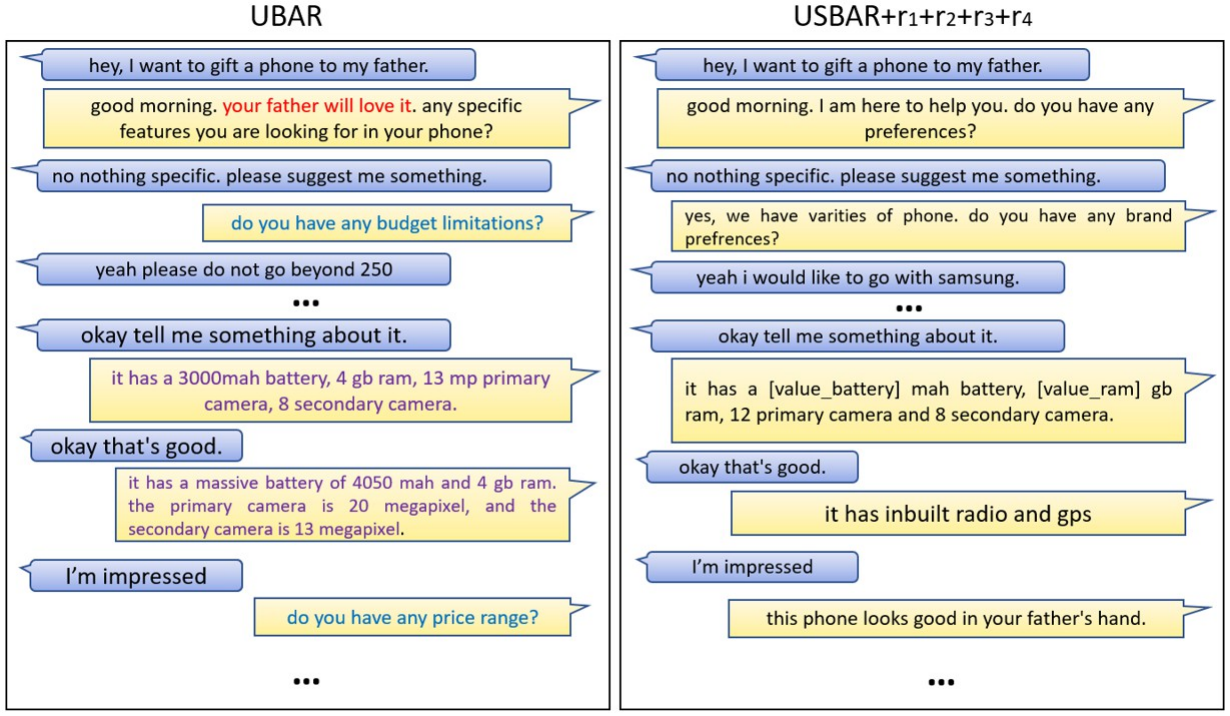
Sample-2.

Here (Fig 12) we are trying to see the models’ performances when the user passes consecutive positive sentiments. In the second reply of UBAR (left image), we can see that the model is asking about the budget of the user and again at the very last reply in that image it is again asking the user about the same. In addition to this, after getting 2nd consecutive positive response, the UBAR model is passing the same information about the battery. Here the model is failing to avoid repetitiveness and the response is not even properly delexicalized. On the other hand, the USBAR (right image) module is trying to pass the battery information after getting the first positive sentiment, then providing information about the radio and gps. So it is clear that the model USBAR+Rewards has improved over UBAR in terms of repetitiveness.

### 10.3 Generated sample 3

**Fig 13:**
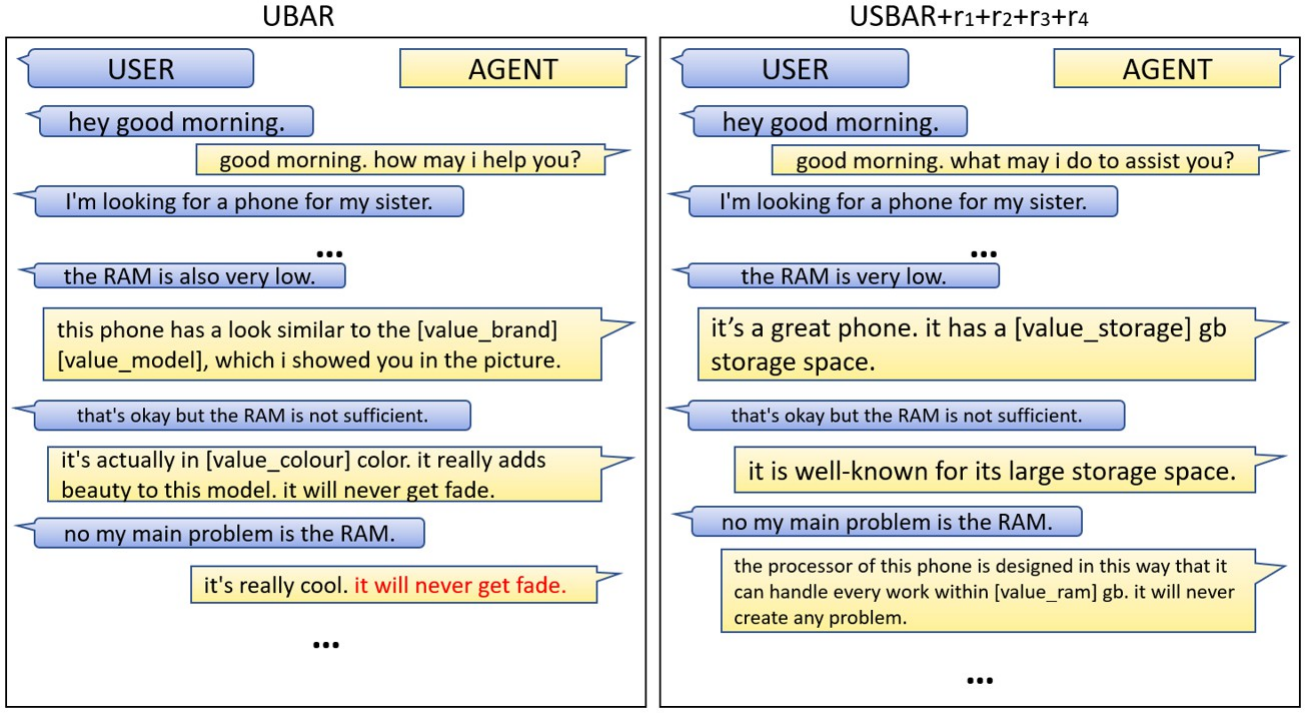
Sample-3.

Again here (Fig 13) we have passed negative sentiments, but this time regarding the RAM. In this scenario, the UBAR module is trying to convince the user with the colour attribute. Again and again the user is expressing his/her dissatisfaction regarding the RAM and in response, every time he/she is receiving something about the colour, which can disgust the user. On the other hand, USBAR+rewards module first highlighted the storage capacity. On the next turn when it realised that this is not working, then it is trying to convince the user by stating about its good processor which can avoid every problem, despite of having a low RAM.

## 11 Conclusion and future works

The current work reports about the development of an end-to-end neural response generation system for sales domain which is having several features:(a) capable of persuading the user in case of goal unavailability situation; if user’s specified goals/specifications are not available in the database, the agent will try to persuade the user regarding some alternative goal; (b) the agent utilizes different persuasion strategies for convincing the user as per the context information; there are 5 different persuasion strategies the agent can follow: emotional appeal, personal appeal, persona based appeal, logical appeal, credibility appeal. The appropriate strategy is selected by the agent based on the context information. To the best of our knowledge, this is the first work on automatic neural response generation in an end-to-end setting for developing a persuasive conversational agent. Moreover, some reinforcement learning based rewards are also introduced with this end-to-end NLG module to improve the model’s performance in terms of less repetitiveness and consistency (both with the context and the generated action). The model is trained using a newly developed data set, namely PPD (personalized persuasive dialogue) and the incorporation of persuasion behaviour is making the model more useful in practical scenarios, specially in sales domain or where the agent needs to do some reservation. We also have improved the domain adaptation power of this model by the inclusion of optimization based meta learning. Results on PPD data set illustrate the impact of meta-learning for domain adaptation and also utility of introducing the RL based reward functions for improving the quality of responses in terms of automatic and human evaluation metrics. For this paper we have concentrated only on a few electronic goods’ sales domain, but we believe this work can be extended to other sub-domains like air conditioner, refrigerator, micro-wave oven etc. and that is also with a low amount of resource. Future work also includes the introduction of multimodality concept in neural response generation system where the system will be capable of extracting slot-value pairs (belief states) from images shown by the user. Moreover, we also aim in developing some models for negotiating chat-bot.
